# Defective Expression and Function of the Leukocyte Associated Ig-like Receptor 1 in B Lymphocytes from Systemic Lupus Erythematosus Patients

**DOI:** 10.1371/journal.pone.0031903

**Published:** 2012-02-15

**Authors:** Barbara M. Colombo, Paolo Canevali, Ottavia Magnani, Edoardo Rossi, Francesco Puppo, Maria Raffaella Zocchi, Alessandro Poggi

**Affiliations:** 1 Department of Internal Medicine, University of Genoa, Genoa, Italy; 2 Department of Hematology, IRCCS-A.O.U-San Martino-IST-National Institute for Cancer Research, Genoa, Italy; 3 Laboratory of Molecular Oncology and Angiogenesis, National Institute for Cancer Research, IRCCS-Azienso Ospedaliera Universitaria (AOU) San Martino, Genoa, Italy; 4 Division of Immunology, Transplant and Infectious Diseases, Scientific Institute San Raffaele, Milan, Italy; Beth Israel Deaconess Medical Center, United States of America

## Abstract

Systemic lupus erythematosus (SLE) is characterized by the production of a wide array of autoantibodies and dysregulation of B cell function. The leukocyte associated Immunoglobulin (Ig)-like receptor (LAIR)1 is a transmembrane molecule belonging to Ig superfamily which binds to different types of collagen. Herein, we have determined the expression and function of LAIR1 on B lymphocyte from SLE patients. LAIR1 expression in peripheral blood B lymphocytes from 54 SLE, 24 mixed connective tissue disease (MCTD), 20 systemic sclerosis (SSc) patients, 14 rheumatoid arthritis (RA) and 40 sex and age matched healthy donors (HD) have been analyzed by immunofluorescence. The effect of LAIR1 ligation by specific monoclonal antibodies, collagen or collagen producing mesenchymal stromal cells from reactive lymph nodes or bone marrow on Ig production by pokeweed mitogen and B cell receptor (BCR)-mediated NF-kB activation was assessed by ELISA and TransAM assay. The percentage of CD20^+^ B lymphocytes lacking or showing reduced expression of LAIR1 was markedly increased in SLE and MCTD but not in SSc or RA patients compared to HD. The downregulation of LAIR1 expression was not dependent on corticosteroid therapy. Interestingly, LAIR1 engagement by collagen or collagen-producing mesenchymal stromal cells in SLE patients with low LAIR1 expression on B cells delivered a lower inhibiting signal on Ig production. In addition, NF-kB p65 subunit activation upon BCR and LAIR1 co-engagement was less inhibited in SLE patients than in HD. Our findings indicate defective LAIR1 expression and function in SLE B lymphocytes, possible contributing to an altered control of B lymphocytes behavior.

## Introduction

The leukocyte associated Ig-like receptor (LAIR) 1 is a transmembrane molecule belonging to the immunoglobulin (Ig) superfamily expressed by all leukocytes [1]–[3]. Its cytoplasmic tail contains immune receptor tyrosine-based inhibitory motifs (ITIM) [1]–[3]. Upon engagement of the extracellular portion of LAIR1, cytoplasmic ITIM bind to the SH2 domain of phosphatases, leading to inactivation of different kinases and down-regulation of cell activation. LAIR1 has been shown to inhibit T and NK cell activation mediated by several activating receptors [4]–[7]. Furthermore, it has been shown that LAIR1 can deliver an inhibiting signal on intracellular free calcium concentration and immunoglobulin (Ig) production induced by the engagement of B cell antigen receptors (BCR) [8], [9]. Collagens are functional ligands for LAIR1 and LAIR1-collagen interaction directly inhibits immune cell activation [10]–[14]. Recently, we have reported that the expression of LAIR1 on chronic B lymphocytic leukemia (CLL) is inversely correlated with disease stage. More importantly, BCR-mediated activation and proliferation of CLL cells was inhibited upon LAIR1 engagement only in those patients expressing LAIR1 at detectable levels [15]. Systemic lupus erythematosus (SLE) is a immune-mediated chronic inflammatory disease characterized by the presence of a wide variety of auto-antibodies produced by dysregulated B cells [16]–[18]. The initiating auto-antigen which is responsible for the disease is not defined yet but it is relevant to determine whether B cells in SLE patients may express a particular phenotype and a consequent behaviour which may be the reason of the observed dysregulation. The aim of this work was to analyze whether B cells of SLE patients may lack LAIR1, thus impairing the interaction of B cells with collagen, the known ligand of LAIR1, and missing an inhibiting signal which regulates activating signals. For comparison, LAIR1 expression was also determined on B lymphocytes from patients suffering from mixed connective tissue disease (MCTD), systemic sclerosis (SSc) and rheumatoid arthritis (RA). We found that B cells from SLE and MCTD, but not RA or SSc, patients are characterized by a lower LAIR1 expression, compared to healthy donors. In a subgroup of SLE patients a large amount of B cells did not express LAIR1 at variance with B cells from healthy donors where a very small subset was LAIR1. More importantly, Ig production initiated through polyclonal mitogen B cell stimulation was inhibited upon LAIR1 ligation by collagen, or collagen-producing lymph node-derived mesenchymal stromal cells (MSC), only in LAIR1^+^ B cells. Likewise, BCR-induced NF-kB p65 nuclear translocation was inhibited by LAIR1 engagement only in B cells expressing high levels of the molecule. These findings would suggest that Ig production in SLE patients may be dysregulated, at least in part, because B cells do not express LAIR1 inhibiting receptor.

## Materials and Methods

### Ethics Statement

Patients suffering from systemic lupus erythematosus (SLE), mixed connective tissue disease (MCTD), systemic sclerosis (SSc), rheumatoid arthritis (RA) and healthy donors (HD) were enrolled in the Vascular Endothelial Growth Factor Study approved by the Ethic Committee of the San Martino Hospital (n.127/06). Bone marrow specimens and lymph node biopsies were obtained during diagnostic procedure to exclude lymphoproliferative diseases (Ethic approval n.0026910/07). According to the ethic committee approval, a written informed consent was obtained from all participants.

### Patients

Patients with SLE (n = 50, table 1, and 4 patients with active disease), MCTD (n = 24), SSc (n = 20) and RA (n = 14) were analyzed for the expression of LAIR1 on peripheral blood B lymphocytes. Diagnoses were performed on the basis of ARA (American Rheumatology Association) criteria for each disease. Most patients (n = 50) were analyzed for LAIR-1 expression and function during a remission phase of the disease (no clinical symptoms characteristic of the disease detectable); four patients with active disease were also studied (SLEDAI>4). Forty aged and sex matched HD were studied as controls. Venous blood samples were drawn and immediately after isolation peripheral blood mononuclear cells (PBMC) were analyzed for the expression of LAIR1 on B cells, identified by the expression of CD20 and surface IgM. Some samples were frozen and then analyzed in functional experiments for Ig production and proliferation in response to BCR engagement or upon stimulation with polyclonal mitogen.

**Table 1:** SLE patients characteristics.

Patient	Diagnosis	age	Sex	Year of disease	Therapy	ESR	ANA titer	CD20^+^LAIR1^−^
pz1	DLE	51	F	16	MP 8 mg/die	28	1∶80	35
pz2	SLE	51	F	27	MP 4 mg/die, Aza 100 mg/die	40	1∶320	30
pz3	SLE	48	F	16	P 5 mg/die, My:2 g/die	37	1∶160	46
pz4	SLE	43	F	16	P 2.5 mg/die My:1 g/die	12	1∶160	14
pz6	SLE	36	F	8	P 5 mg/die, MTX:7.5 mg/w, HC:200 mg/die	8	1∶160	23
pz7	SLE	38	F	10	MP 16 mg/die, My:2 g/die	24	1∶640	18
pz10	SLE	71	F	22	P 5 mg/die, Aza:100 mg/die	58	1∶80	47
pz11	SLE	45	F	6	P 12.5 mg/die; HC 200 mg/die, MTX 10 mg/w	14	1∶160	25
pz12	SLE	36	M	2	P10 mg/die, Aza:100 mg/die, HC 200 mg/die	30	1∶640	32
pz16	SLE	38	F	20	MP 40 mg/die, Aza 100 mg/die, CsA 150 g/die	5	1∶80	24
pz17	SLE	67	F	31	MP 8 mg/die, Aza:100 mg/die, HC 200 mg/die	35	1∶160	35
pz18	SLE+APS	36	F	4	MP 40 mg/die	30	1∶160	26
pz19	SLE	68	M	19	MP 8 mg/die, CsA 100 mg/die	40	1∶160	55
pz22	SLE	44	F	22	MP 4 mg/die	14	1∶160	70
pz24	SLE	44	M	11	P 12.5 mg/die, Aza 100 mg/die	12	1∶160	18
pz28	SLE	65	F	39	MP 4 mg/die	32	1∶160	40
pz33	SLE	46	F	27	D 12 mg/die, Aza 50 mg/die	50	1∶160	28
pz35	SLE	48	F	2	P 5 mg/die, My 1.5gr/die	34	1∶160	28
pz42	DLE	46	F	3	F (topic)	18	1∶320	29
pz44	SLE	27	F	3	MP 8 mg/die, My 2.0gr/die, HC 200 mg/die	17	1∶320	22
pz47	SLE	22	F	1	P 2.5 mg/die, My 200 mg/die,	13	1∶320	70
pz52	SLE	39	M	17	MP 16 mg/die	15	1∶160	17
pz53	SLE	51	F	21	D 6 mg/die, My 1.5gr/die, HC 200 mg/die	10	1∶160	61
pz54	SLE	57	F	24	MP 8 mg/die, My 1.0gr/die	16	1∶160	11
pz55	SLE	35	F	15	MP 8 mg/die, HC:200 mg/die	30	1∶160	31
pz59	SLE	65	F	15	MP 8 mg/die My 1.5gr/die, HC:200 mg/die	24	1.160	24
pz61	SLE	22	F	1	P 2.5 mg/die HC 200 mg/die	13	1∶80	14
pz65	SLE	37	F	23	MP 4 mg/die	20	1∶160	27
pz66	SLE	32	F	13	MP 8 mg/die, HC 200 mg/die	8	1∶160	6
pz68	SLE	37	F	15	MP 40 mg/die, Cy 500 mg/die, CsA 200 mg/die	40	1∶80	39
pz73	SLE	65	F	4	P 2.5 mg/die	16	1∶80	20
pz76	SLE	31	M	7	D 6 mg/die, My 2gr/die, HC 200 mg/die	21	1∶640	58
pz77	SLE	38	F	3	MP 8 mg/die, HC 200 mg/die	15	1∶160	13
pz78	SLE	55	F	30	D 6 mg/die, HC:200 mg/die	20	1∶80	36
pz80	SLE	36	F	18	P 5 mg/die, HC 200 mg/die, MTX 10 mg/w	8	1∶160	66
pz81	SLE	39	F	18	P 5 mg/die, HC 200 mg/die, CsA 100 mg/die	11	1∶640	50
pz82	SLE	45	F	19	MP 8 mg/die, HC 200 mg/die	28	1∶160	45
pz14	SLE	34	F	12	HC 200 mg/die	18	1∶160	16
pz20	SLE	49	F	18	none	20	1∶160	60
pz37	SLE	65	F	25	MTX 7.5 mg/w	15	1∶80	31
pz38	SLE	39	F	16	My 2gr/die	10	1∶80	10
pz39	SLE	39	F	10	My 1gr/die	40	1∶160	14
pz43	SLE	43	F	10	HC 200 mg/die	20	1∶160	22
pz48	SLE+APS	63	F	1	MTX 7.5 mg/w	21	1∶160	80
pz49	SLE	39	F	17	none	30	1∶160	33
pz50	SLE+APS	47	F	10	none	8	1∶80	18
pz51	SLE	63	F	13	none	5	1∶160	18
pz56	SLE	44	F	16	HC 200 mg/die	16	1∶160	3
pz60	SLE	37	F	4	HC 200 mg/die	12	1∶640	6
pz71	SLE	45	F	5	none	16	1∶80	55

Diagnoses were based on ARA criteria. SLE: Systemic Lupus Erythematosus; APS: Anti-Phospholipid Syndrome; DLE: Discoid Lupus Erythematosus; ESR: Erythrocyte Sedimentation Rate in mm/h (first hour); ANA: Anti-Nuclear Antibodies. nd: not done; CD20^+^LAIR1^−^ B cells determined by immunofluorescence in each patients are also shown. MP: methyl-prednisolone; P: prednisone; D: deflazacort; F: fluocortolone; Aza: azathioprine; HC: hydroxy chloroquine; My: mycophenolate mofetil; CsA: cyclosporin A; Cy: cyclophosphamide. Data of ESR, ANA and percentages of CD20^+^LAIR1^−^ cells are referred to the same sample of venous blood for each patient.

#### Monoclonal antibodies (mAbs) and reagents

Anti-human LAIR1 (NKTA255, IgG1), and anti-CD3 (JT3A, IgG2a) mAbs were obtained as described [19]. Anti-SH2 (CD105, IgG1), anti-SH3 (CD73, IgG2b), mAbs producing hybridomas were purchased from the American Type Culture Collection (ATCC, Manassas, VA). Anti-HLAABC mAb (clone 3A3, IgM) mAb was kindly provided by E. Ciccone (University of Genoa). Antiprolyl-4-hydroxylase mAb (clone 5B5, IgG1) was purchased from Dako (Glostrup, Denmark). Anti-human CD146 (clone P1H12, IgG1) and unrelated isotype matched control mAbs were from BD Pharmingen, anti-CD90 (clone Thy-1A1, IgG2a) and anti-HMGB1 (clone 115603, IgG2b) mAbs were from R&D System Inc. (Minneapolis, USA) and anti-CD27 mAb (clone LT27, IgG2a) was from Serotec (Oxford, UK). Anti-BSP (bone sialoprotein, clone LN-1, IgM) was from Abnova (Abnova GmbH, Germany). Anti-ALP (alkaline phosphatase, clone 8B6, IgG2a), anti-vimentin (clone V9, IgG1), anti-collagen I (clone COL-1, IgG1) and anti-collagen III (clone FH-7A, IgG1) mAbs, pokweed mitogen (PWM), acetoxymethyl-ester of fura 2 (Fura2-AM), collagen I, II, III and goat anti-human μ chain specific FITC-conjugated polyclonal or purified monoclonal antibody were from Sigma Chemicals Co. (St. Louis, MO, USA), while macrophage-activating lipopeptide-2 (MALP2) was from Enzo Life Sciences (Loerrach, Germany). Complete medium for culture of PBMC or B cells was composed of RPMI1640 with 10% of fetal calf serum supplemented with 1% antibiotics (penicillin and streptomycin) and 1% L-glutamine (Biochrom, Berlin, Germany).

#### Isolation of peripheral blood mononuclear cells and B cells

PBMC were isolated from venous blood samples by separation on Ficoll-Hypaque as described previously [20]. In some experiments, B lymphocytes from HD were selected by negative selection. Briefly, purified B cells were obtained after depletion of monocytes by plastic adherence and addition of red blood cells to PBMCs at 30∶1 ratio, with the RosetteSep B cell enrichment kit (StemCell Technologies, Vancouver, Canada) and were >98% pure as assessed by CD20 staining [20]. In some experiments LAIR1^−^ B cells (>95% LAIR1^−^) from some SLE patients were obtained from the whole B cell population after negative selection using magnetic beads coated with GAM (Dynal, Invitrogen srl).

#### Immunofluorecsence assay

PBMC samples from SLE, MCTD or SSc patients and HD were stained with the indicated mAbs followed by goat-anti-mouse isotype specific antibody conjugated with the reported fluorochromes and then run on a Cyan ADP cytofluorimeter (Beckman-Coulter) equipped with an argon ion laser exciting FITC and PE at 488 nm or an He-Neon far red laser exciting alexafluor647 isotype specific goat-anti mouse (GAM). Results are expressed as log mean fluorescence intensity (MFI) arbitrary units (a.u.) vs number of cells and analyzed with the Summit v4.3 computer program (Beckman- Coulter). Results of double or triple immunofluorescence assays are expressed as log red MFI vs log green MFI or vs log far red MFI. Samples for confocal microscopy were analyzed by FV500 (Fluoview confocal Laser Scanning Microscope System, Olympus Europe GMBH, Hamburg, Germany) after observation with PlanApo 40× NA1.00 or 60× NA1.40 oil objectives and data analyzed with FluoView 4.3b computer program (Olympus). Each image has been taken in sequence mode to avoid cross-contribution of each fluorochrome. Results are shown in pseudocolor.

### Immunoglobulin (Ig) production assay

PBMC isolated from SLE or HD were incubated with PWM (5 µ g/ml) for 5 d at 37°C in complete medium. Then culture supernatants (SNs) were harvested and analyzed for the presence of human Ig of M, G and A isotype with a specific ELISA kit purchased from Bethyl (Bethyl Laboratories Inc, Montgomery, USA). The amount of Ig present in SNs was calculated with a standard curve. To evaluate the inhibiting signal delivered by LAIR1 on Ig production, parallel samples were preincubated with anti-LAIR1 mAb for 30 min at 4°C, washed and incubated for 5 d with PWM and goat anti-mouse immunoglobulin (GAM Ig) to achieve LAIR1 oligomerization (LAIR1-XL). Other samples were pretreated with an unrelated mAb matched for isotype as control. In some experiments Ig production was evaluated in SN of PBMC cultured for 5 d on plastic Petri dishes coated with a mixture of collagen I, II and III (10 µ g/ml) and with or without the F(ab′)_2_ fragment of anti-LAIR1 mAb prepared as described [21]. In preliminary experiments, we determined that the use of F(ab′)_2_ does not induce appropriate engagement of LAIR1 for inducing inhibiting signal but it was efficient in blocking ligation of LAIR1 with collagen.

### Human MSC-lymphocytes co-culture experiments

Human MSC were obtained as described [22] from 6 biopsies of reactive lymph nodes (LMSC) isolated during diagnostic procedure to exclude lymphoproliferative diseases. LMSC displayed a fibroblast-like morphology and expressed CD73, SH4, CD105, CD44, β1-integrin/CD29, ICAM1/CD54, CD146, HLA-I, prolyl-4-hydroxylase (the enzyme which catalizes the hydroxylation of the aminoacid proline), alkaline phoshatase (ALP), collagen, vimentin, bone sialoprotein BSP), and osteopontin virtually on all cell populations analyzed. LMSC did not express CD45, CD31, CD34, CD33, CD3, CD2, CD16, CD14, ICAM2, ICAM3, CD80, CD86, CD83, and HLA-DR (not shown). The surface phenotype of LMSC was stable along culture period (2–3months, 25 doubling time). LMSC were co-cultured with 10^5^PBMC or with purified B cells at the LMSC∶PBMC/B ratio of 1∶4 in medium alone or in the presence of PWM in flat-bottomed microplates in 200 µ l and Ig content of supernatant was analyzed as described above on day 5 of culture. This LMSC∶PBMC/B ratio was chosen because the inhibition of Ig production was evident only at 1∶2, 1∶4 and 1∶8 LMSC∶PBMC/B ratio as previously reported [23]. At 1∶16 and 1∶32 LMSC∶PBMC/B ratio we could not detect any inhibiting effect on Ig production [24]. To determine the role of MSC-PBMC/B contact in regulating B cell Ig production, in some experiments, PBMC were seeded on Millicell transwell (TW) with 0.3 µ m pores (Millipore Corporation, Billerica, MA) put in 24-well plates with LMSCs seeded on the bottom to avoid LMSC-PBMC/B cell contact.

#### Calcium mobilization assay

Purified B cells were loaded with fura-2AM (1 µ M, Sigma) for 1 h at 37°C, placed in a quartz 2-ml cuvette and maintained at 37°C by a thermostatically controlled water bath. Fura2-AM was exited at 334 and 380 nm, emitted light was filtered at 510 nm and fluorescence was monitored with an LS-50B spectrofluorimeter (Perkin-Elmer, Pomona, CA). [Ca^2+^]_i_, calculated as described [19], was measured upon cross-linking of sIgM (IgM-XL), obtained with the specific purified mAb followed by (GAM Ig), or after sIgM and LAIR1 co-engagement (sIgM-LAIR1-XL).

#### NF-kB Transcription factor activation assay

NF-kB activation was evaluated by the TransAM Assay Kit in nuclear extracts obtained using the Nuclear Extract Kit (Active Motif, Rixensart, Belgium) from B lymphocytes from HD or SLE patients incubated for 12 h alone or after cross-linking of sIgM and/or LAIR1, in the presence or absence of LAIR1pep or LAIR1mock. The SHP-1-binding peptide, spanning the Tyr233-Tyr263 region of LAIR1 cytoplasmic tail [15], VTYAQLDHWALTQRTARAVSPQSTKPMAESITYAAV (LAIR1pep), or the scrambled peptide VAATDRWSYKPQETQALSHAYRMALTIVATSAQLVT (LAIR1pepM) were obtained from PRIMM (Milan, Italy) and used at the concentration of 10 µ g/ml, as reported [15]. The time point was chosen on the basis of preliminary kinetics experiments in order to detect optimal NF-kB activation. TransAM NF-kB Kits is a 96-well plate with immobilized oligonucleotide containing the NF-kB consensus site (5′-GGGACTTTCC-3′) that specifically binds to the active form of NF-kB contained in cell extracts. Primary antibodies directed against an epitope on p65 subunit, that is accessible only when NF-kB is activated and bound to its DNA target, are then added, followed by HRP-conjugated secondary antibody and reaction developed by the specific substrate contained in the commercial kit. Plates were then read with and ELISA reader and results expressed as OD_450 nm_. Percent of activation of p65 NF-kB activation was determined with the following formula: nucleus/total amount of p65 NF-kB ×100.

### Statistical analysis

Statistical analysis was performed using two tales Student's t test at 95% confidence or Pearson coefficient r with GraphPad Prism 4.0.

## Results

### SLE patients display higher percentage of CD20^+^ B cells lacking LAIR1 than healthy donors

**Figure 1:**
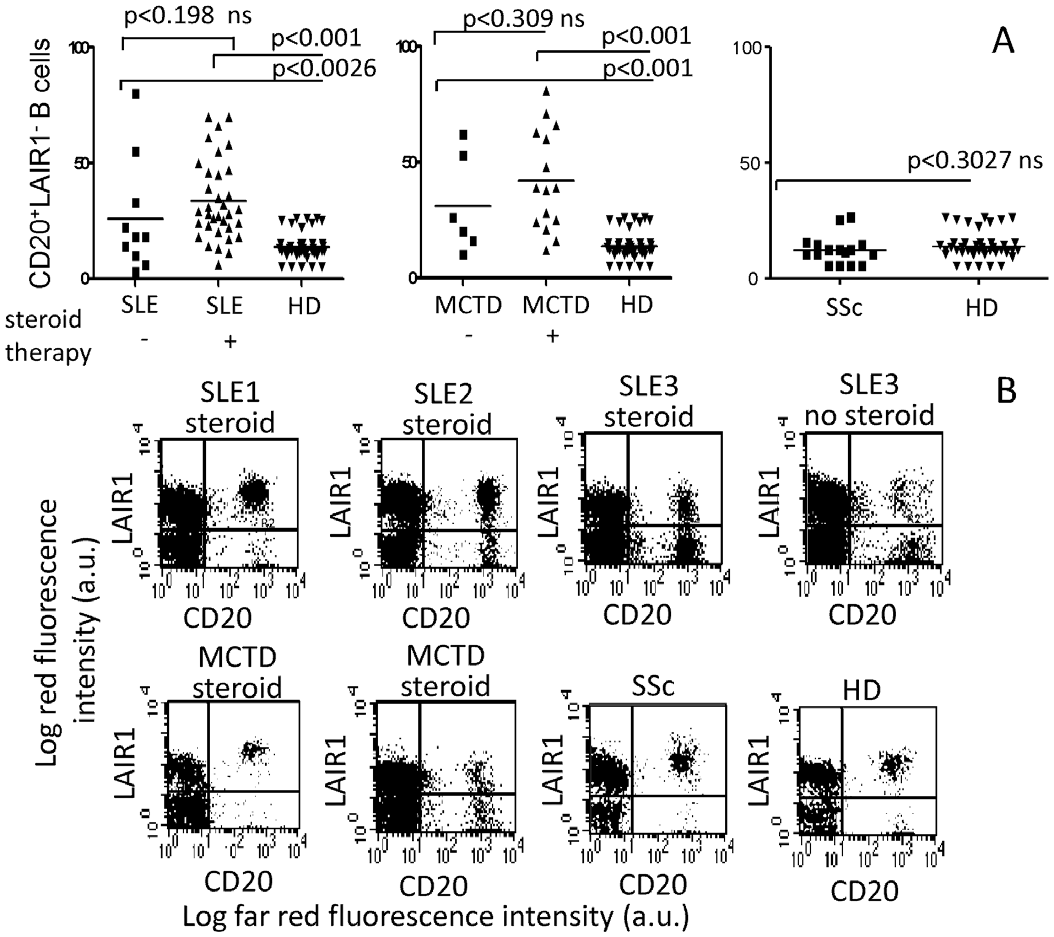
Expression of LAIR1 on PBMC from SLE or RA or SSc patients and HD. A. Peripheral blood mononuclear cells (PBMC) were isolated from SLE (n = 50) or RA (n = 14) or SSc (n = 20) patients and stained with anti-LAIR1 mAb and anti-CD20 mAb followed by goat anti-isotype specific GAM conjugated with either PE or Alexafluor647 and goat anti-human sIgM-FITC polyclonal antibody. Results are expressed as CD20^+^LAIR1^−^ B cells gated on sIgM^+^ cells. Surface IgM^+^ B cells were 95–100% of all B cells in PBMC of any population tested. The percentages of CD20^+^LAIR1^−^ cells present in PBMC of healthy donors (HD) is shown for comparison in each panel (n = 40). A, right panel: Patients suffering from SLE were subdivided in two groups (with or without steroid therapy). The statistical significance of results is shown in each panel. B. Some representative examples of expression of CD20 and LAIR1 on PBMC of SLE in remission phase (SLE1 and SLE2) or in acute phase (SLE flare) or RA or SSc patients and HD. Results are expressed as Log far red fluorescence intensity vs Log red fluorescence intensity. Each dot plot is subdivided into four quadrants representing CD20^−^LAIR1^+^ (upper left) or CD20^+^LAIR1^+^ (upper right) or CD20^−^LAIR1^−^ (lower left) or CD20^+^LAIR1^−^ (lower right) cells respectively.

First, we analyzed the expression of LAIR1 receptor on peripheral B cells of SLE (n = 50, table 1), AR (n = 14), SSc (n = 20), or MCTD (n = 24) patients and HD (n = 40), by staining PBMC with anti-LAIR1, anti-CD20 mAb followed by PE or Alexafluor647-conjugated anti-isotype specific GAM and sIgM-FITC conjugated antibodies. Results shown in figure 1A are gated on sIgM^+^ B cells. The percentage of CD20^+^ B cells which did not express LAIR1 was markedly increased in SLE patients compared to HD (40±10% vs 10±5%, p<0.001) and to AR patients (p<0.002) or SSc patients (p<0.002, fig. 1A left panel). Furthermore, we found that the statistical significance of the increase of CD20^+^LAIR1^−^ cells in SLE (p<0.001) patients treated with corticosteroids was similar to that of untreated patients while there was no a significant statistical difference between steroid treated and untreated patients (p<0.198) (fig. 1A, right panel). In addition, no correlation between the percentage of CD20^+^LAIR1^−^ B cells and cortisol equivalent dose administered to each patient was found (r = −0.185). Also, in MCTD patients an increment of CD20^+^LAIR1^−^ B cells was detectable (p<0.001 vs HD, not shown) and again no statistical differences were found between steroid treated and untreated patients (p<0.309, not shown).

Some representative examples of the expression of LAIR1 on unseparated PBMC from SLE, RA, or SSc patients and HD are shown in fig. 1B. Of note, the MFI of LAIR1 on CD20^+^LAIR1^+^ B cells was strongly decreased in SLE patients (MFI = 330±120 arbitrary units, a.u.) as compared to HD (MFI = 815±120a.u.). Two groups among SLE patients on the basis of the pattern of LAIR1 expression could be defined. The first group (SLE1, n = 17) displayed a pattern of LAIR1 expression similar to HD (fig. 1B upper panels, first dot plot, CD20^+^LAIR1^−^ B cells >20%). The second group, that includes the majority of SLE patients (SLE2, n = 43), displayed a lower MFI of LAIR1 expression and a higher percentage of CD20^+^LAIR1^−^ B cells than HD (fig. 1B, upper panels, second dot plot vs lower panels first dot plot, CD20^+^LAIR1^−^B cells 60±10% vs 10±5%, p<0.001) or AR patients (fig. 1B, lower panels, second dot plot). Finally, LAIR1 expression on B cells in SLE patients with active disease has been analyzed in 4 cases (one shown in fig. 1B, upper panels, third dot plot): in these cases MFI was superimposable to that of SLE in remission phase (MFI = 300±100 a.u. vs 330±120 a.u.) while the percentage of CD20^+^LAIR1^−^ cells was 30±10%.

**Figure 2:**
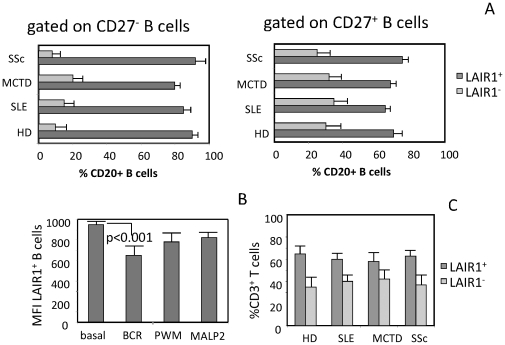
Expression of LAIR1 on naïve and memory B cells, effect of B cell activation on LAIR1 expression and LAIR-1 expression on T cells. Panel A. Percentage of LAIR1^+^ (black bar) or LAIR1^−^ B (grey bar) cells in naïve (CD27^−^) or memory (CD27^+^) cell subsets. Results are determined by triple immunofluorescence assay using specific mAbs to CD20, LAIR1 and CD27 followed by isotype specific GAM conjugated with Alexafluor674 (CD20) or PE (LAIR1) or Alexafluor488 (CD27) on HD or SLE or MCTD or SSc patients. Panel B. Effect of B cell activation on LAIR1 expression. Highly purified healthy B cells were stimulated with sIgM or PWM or MALP2, as indicated, and expression of LAIR1 and CD20 was analyzed as in A. Results are representative of seven independent experiments. Panel C. Expression of LAIR1 on T cells from healthy donors (HD), SLE, MCTD and SSc patients. PBMC were stained with anti-CD3 (JT3A, IgG2a) and anti-LAIR1 (IgG1) mAb followed by anti-isotype specific GAM conjugated with Alexafluor647 or PE respectively. Results are expressed as percentages of CD3^+^ LAIR1^+^ (dark grey bar) or CD3^+^LAIR1^−^ (light grey bar) T cells.

We analyzed whether the lower expression of LAIR1 on B cells of SLE patients was related to an imbalance between naive and memory B cells. Thus, we labeled HD, SLE and MCTD PBMC with anti-CD27, anti-LAIR1 and anti-CD20 mAb to identify CD20^+^CD27^−^ as naïve or CD20^+^CD27^+^ as memory B cells respectively. We found that LAIR1 was expressed by most if not all CD20^+^CD27^−^ naïve B cells in any kind of patients or HD; likewise, more than 70% of CD20^+^CD27^+^ memory B cells were LAIR1^+^ in SLE, MCTD, SSc and HD, suggesting no evident imbalance of naïve and memory B cell subsets in patients analyzed (fig. 2A) The decreased expression of LAIR1 in SLE and MCTD patients may be related to the activation of B cells as previously reported (8). Thus, we analyzed whether LAIR1 expression was effectively down-regulated during activation of B cells. To this aim, highly purified (>95% CD20^+^) healthy B cells were cultured with different polyclonal B cell stimuli as anti-IgM antibodies (anti-BCR), or PWM or MALP2 by adding IL2 on d2 and LAIR1 expression was evaluated on d6 of culture. PWM or MALP2 were used to mimic B cell stimulation through other receptor than BCR. The effective activation of peripheral B cells was assessed by the increase of expression of CD38 and neo-expression of CD25 antigens (not shown). A statistically significant reduction of fluorescence intensity of LAIR1 expression (p<0.001) was found on d6 after B cell stimulation through BCR compared to ex-vivo B cells (MFI = 815±120a.u. on d0 vs MFI = 650±90a.u. after anti-BCR stimulation, fig. 2B). Stimulating B cells with PWM or MALP2 (fig. 2B), a slightly reduction of LAIR1 expression was detected. It is of note that the percentage of CD20^+^LAIR1^−^ B cells found in a given donor was not increased by any of the stimuli used (not shown). Further experiments were performed in order to clarify whether, besides B cells, also T lymphocytes showed a decreased LAIR1 expression in SLE patients. We found that LAIR1 was expressed on T lymphocytes of SLE and MCTD patients at levels comparable to those detected on T cells from HD and SSc patients (not shown); in addition, the percentages of LAIR1^+^ and LAIR1^−^ T cells present in peripheral blood were similar in SLE, MCTD, SSc and HD (fig. 2C).

### LAIR1 oligomerization induces the down-regulation of Ig production on healthy B lymphocytes: this effect is related to LAIR1 expression in SLE patients

**Figure 3:**
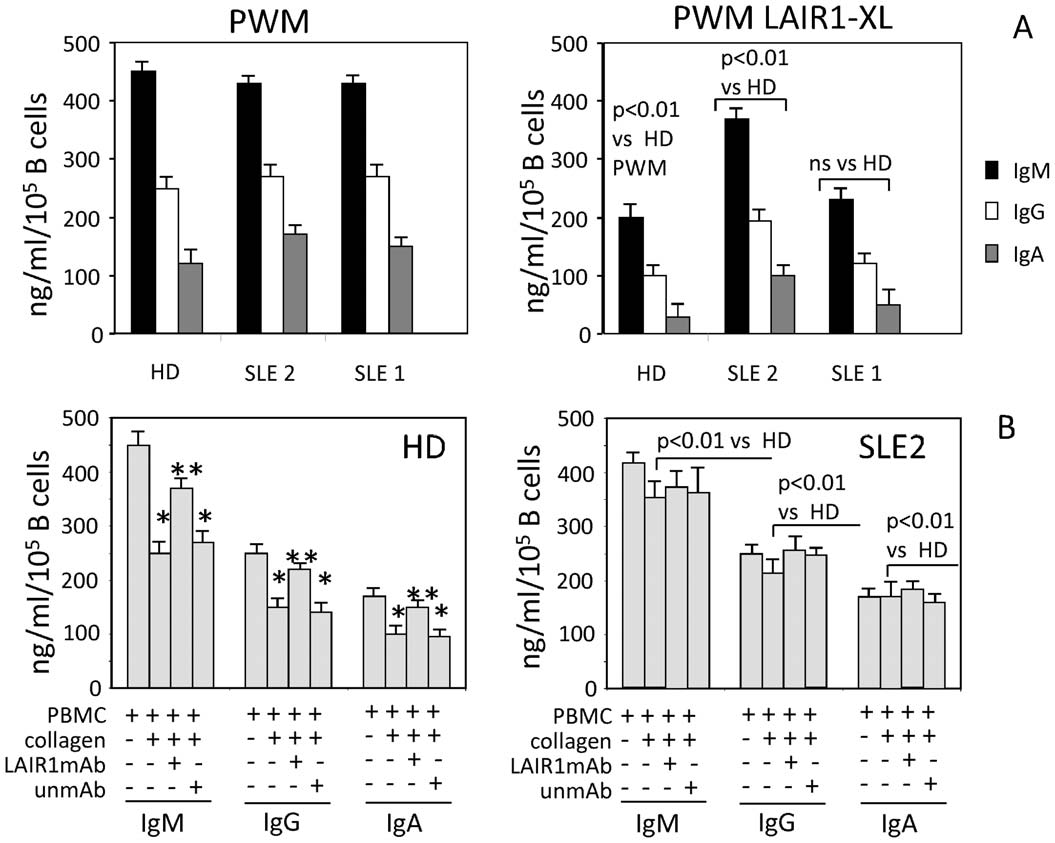
Production of Ig by PBMC from SLE patients stimulated with pokeweed mitogen: regulation through the engagement of LAIR1 and collagen. A. Immunoglobulin production of the indicated isotype (A or G or M) from healthy donors (HD) or SLE patients was analyzed with specific ELISA kit in cell culture supernatant after stimulation for 5 d of PBMC with pokeweed mitogen alone (PWM) (left) and upon cross-linking of LAIR1 (PWM LAIR1-XL) (right). Results are expressed as ng/ml/10^5^ CD20^+^ B cells. Ig production of PBMC from HD (n = 25) is shown for comparison. In the right panel the statistical significance was shown between HD+PWM and HD+PWM-LAIR1-XL, or HD and SLE2 or SLE1 groups in PWM-LAIR1-XL culture condition. Panel B. PBMC of HD, (n = 8, left) or SLE patients (n = 10 from SLE2 group, right) were incubated for 5 d on collagen coated plates. Then SN were analyzed for the presence of the human IgM, IgG and IgA by ELISA. In some experiments, F(ab′)_2_ of anti-LAIR1 mAb (5 µ g/ml) to compete with the interaction of surface LAIR1 and collagen or an unrelated mAb matched for the isotype as control mAb (5 µ g/ml) was added at the onset of cell culture. Results are expressed as ng/ml/10^5^ CD20^+^ B cells as mean±SD. * p<0.001 vs basal production of Ig. **p<0.001 vs Ig production of PBMC on collagen coated plates. In the right panel is indicated the statistical significance of Ig production in the culture condition PBMC+collagen in SLE2 patients vs HD.

We then analyzed whether the ligation of LAIR1 could affect Ig production in SLE patients and whether this effect was related to the level of LAIR1 antigen expression on B cells. To this aim, PBMC isolated from SLE patients were stimulated for 5 d with the polyclonal stimulus PWM and SNs were collected and analyzed for the presence of human Ig by ELISA. Parallel samples of PBMC from HD were stimulated with PWM as control. Healthy B cells produced Ig upon stimulation with PWM (fig. 3A, left panel) and LAIR1 oligomerization strongly inhibited Ig production (fig. 3A, right panel). Indeed, IgM or IgG or IgA release in culture SN was reduced by 50–60% or 60–70% or 80–90% respectively by the engagement of LAIR1. Upon stimulation with PWM, PBMC from SLE patients produced amounts of IgM, IgG and IgA at similar levels as PBMC from HD (fig. 3A, left). Of note, inhibition of PWM-induced Ig production following LAIR1 engagement in PBMC of SLE patients with B cells expressing low levels of LAIR1 or high fraction of LAIR1^−^ (SLE2 group) was markedly reduced compared to that found in healthy PBMC. Indeed, Ig production in SLE2 group was 85% of that found without LAIR1 engagement (fig. 3A) with a statistically significant difference between SLE2 and HD (p<0.01, fig. 3A, right panel). On the other hand, LAIR1-mediated inhibition of Ig production by SLE B cells expressing normal LAIR1 levels (SLE1 group) was comparable to that found in HD. Finally, a strong direct correlation between the % of LAIR1^+^ B cells and the % of LAIR1-mediated inhibition on PWM-induced Ig production was found (r = 0.94 or r = 0.91 or r = 0.87 for IgM or IgG or IgA respectively).

### LAIR1-collagen interaction do not inhibit Ig production in SLE patients expressing low levels of LAIR1 or with a large proportion of LAIR1^−^CD20^+^ B cells

It is relevant to analyze the effects of engagement of LAIR1 in SLE patients upon interaction with its natural ligand that is collagen [10]–[13]. Thus, in a first series of experiments, we analyzed whether the lack or low expression of LAIR1 on B cells of SLE patients could lead to a decrease of the inhibiting signal delivered to B cells upon LAIR1-collagen interaction. In order to test the LAIR1-mediated inhibiting signal, we evaluated Ig production by PWM stimulated B cells; we chose this kind of stimulation as it was polyclonal and not necessarily dependent on interaction with BCR. We found that the production of Ig was reduced by culturing healthy B cells on collagen-coated plates. Indeed, IgM or IgG or IgA production on collagen was about 50% or 30% or 40% respectively of that in absence of collagen (fig. 3B, left panel). This inhibiting effect was partially reverted (about 50% recovery) by blocking LAIR1-collagen interaction with the F(ab′)_2_ fragment of LAIR1 mAb. No effect was exerted by an isotype matched unrelated mAb (fig. 3B).

As shown in Fig. 3B (right panel), the collagen-LAIR-1-mediated inhibition of Ig production was almost not detected (about 10% of inhibition) in SLE2 patients which express low levels of LAIR1 and a large proportion of LAIR1^−^CD20^+^ B cells. Indeed, the production of IgM, IgG and IgA was similar to that observed in the absence of collagen (e.g for IgM: 410±15 ng/ml in PBMC vs 350±25 ng/ml in PBMC cultured on collagen). Importantly, the production of IgM. IgG and IgA in the presence of collagen in SLE2 patients was statistically significant increased compared to that found in HD under the same culture conditions (p<0.01).

### The interaction of B cells with collagen-producing MSC inhibits Ig production through the involvement of LAIR1 in HD: this effect is defective in SLE B cells

**Figure 4:**
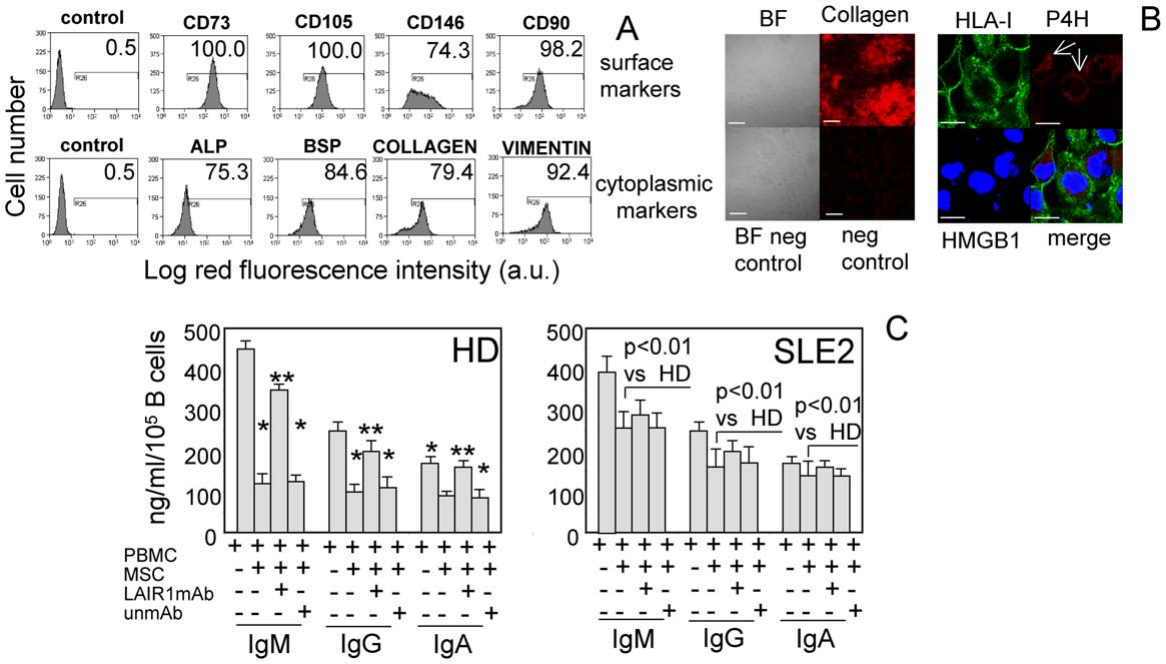
Collagen-producing LMSC regulate Ig production upon engagement of LAIR1: this effect is defective in SLE patients. A, LMSC from a representative reactive lymph node were surface stained with the indicated mAbs (first row) followed by PE-conjugated anti-isotype specific goat anti-mouse antiserum. Control: cells stained with an unrelated mAb followed by GAM as negative control. Second row: LMSC were cytoplasmic stained after fixation and permeabilization with mAbs to the indicated molecules (ALP: alkaline phosphatase, BSP: bone sialoprotein, collagen or vimentin) followed by PE-conjugated GAM. Results are expressed as log red fluorescence intensity vs number of cells. In each panel are indicated the percentages of positive cells above the horizontal bar set on negative control (first subpanel on the left of each row). B. left: bright field (BF) of LMSC from a representative reactive lymph node (upper left), staining with anti-collagen mAb (upper right, red) without cell permeabilization and the respective negative controls (BF neg control, neg control). B right: staining of LMSC with anti-HLA-I (surface, green), anti-prolyl-4-hydroxylase (P4H, cytoplasmic, red) and anti-HMGB1 mAb (nucleus, blue) analyzed by confocal microscopy. Merge analysis is also shown. 400× (left), 600× (right) magnification. White Bars: 10 µ m; reactivity for collagen is disposed in large and concentrated regions (upper left); the white arrows indicate the intracytoplasmic reactivity for P4H (upper right). C. PBMC of healthy donors (HD, n = 7, left) or SLE patients (n = 9 from SLE2 group, right) were incubated for 5 d on collagen-producing mesenchymal stromal cells (MSC) from reactive lymph node coated plates. Then SN were analyzed for the presence of the human IgM, IgG and IgA by ELISA. In some experiments, F(ab′)_2_ of anti-LAIR1 mAb (5 µ g/ml) to compete with the interaction of surface LAIR1 and collagen-producing MSC or an unrelated mAb matched for the isotype as control mAb (5 µ g/ml) was added at the onset of cell culture. Results are expressed as ng/ml/10^5^ CD20^+^ B cells as mean±SD. * p<0.001 vs basal production of Ig. ** p<0.001 vs Ig production on LMSC coated plates. In the right panel is indicated the statistical significance of Ig production in the culture condition PBMC+LMSC in SLE2 patients vs HD.

We also determined whether collagen producing cells can deliver an inhibitory signal on Ig production through LAIR1 engagement. Indeed, it is commonly accepted that the production of Ig can happen in lymph nodes and in SLE disease the interaction between lymph node-derived mesenchymal stromal cells (LMSC) and B cells can be considered as a pathophysiological conditions where the lack of expression of LAIR1 can contribute to altered Ig production because the interaction of LAIR1 and collagen produced by LMSC is impaired. To this aim, we first established appropriate culture conditions to expand in vitro LMSC from lymph nodes, then we determined whether they can produce collagen and finally LMSC were cultured with healthy B cells or B cells from SLE patients, stimulated with PWM and SNs were analyzed for Ig content on d5. The surface and cytoplasmic phenotype of LMSC used in these experiments is shown in figure 4A. LMSC were typical fibroblast-like cells expressing surface markers of mesenchymal stromal cells as CD73, CD90, CD105 and CD146 (fig. 4A) but lacking markers characteristic of leukocyte including CD34, CD33, CD80, CD86 and HLA-DR (not shown). Importantly, LMSC expressed cytoplasmic alkaline phosphatase, bone sialoprotein, vimentin and collagen as shown by immunofluorescence and FACS analysis (fig. 4A) on permeabilized LMSC and could release collagen in the extracellular microenvironment as shown by immunofluorescence and confocal microscopy analysis (fig. 4B). Indeed, collagen outside of cells appeared to be heterogeneously distributed in large amasses (fig. 4B, left). In addition, LMSC expressed into the cytoplasm prolyl-4-hydroxylase (fig. 4B, right), the enzyme involved in the hydroxylation of collagen. All these features strongly support that LMSC can be considered as cells able to synthetize, process and secrete collagen in the extracellular milieu where it can interact with LAIR1 expressed on B cell surface and deliver an efficient inhibiting signal. Indeed, SN harvested from co-cultures of healthy B cells stimulated with PWM in the presence of LMSC contained a lower amount of Ig than that found in B cell cultured alone; the optimal LMSC∶B cell ratio at which an evident reduction of Ig production was detected was the 1∶4 LMSC∶B cell ratio (fig. 4C) (30% or 40% or 50% of IgM or IgG or IgA of the amount produced without LMSC). Although not shown, this inhibiting effect was evident only when B cells were in contact with LMSC; indeed, no inhibition of Ig production was detected when B cells were co-cultured with LMSC separated by a transwell Millicell chamber. Importantly, the addition of the F(ab′)_2_ fragment of anti-LAIR1 mAb, thus conceivably impairing LAIR1-collagen interaction, partially restored Ig production when B cells were in contact with LMSC (fig. 4C) (90% or 95% or 98% of IgM or IgG or IgA of the amount produced without LMSC), suggesting that, also in this experimental system, LAIR1 surface molecule interacting with collagen can deliver an inhibiting signal on B cells. On the other hand, the Ig production in the SLE2 group of patients in the presence of LMSC was really stronger than that of healthy B cells (270 ng/ml vs 130 ng/ml in SLE2 vs HD). Indeed, in SLE2 group, the amount of IgM or IgG or IgA produced with MSC was 70% or 71% or 80% of that produced without LMSC respectively. The addition of anti-LAIR1 mAb did not lead to a statistically significant increment of Ig production, suggesting that LAIR1 inhibiting signal is not operating in SLE2 patients.

### LAIR1-induced regulation of BCR-mediated calcium mobilization and nuclear translocation of NF-kB p65 subunit is defective in B cells from SLE patients

**Figure 5:**
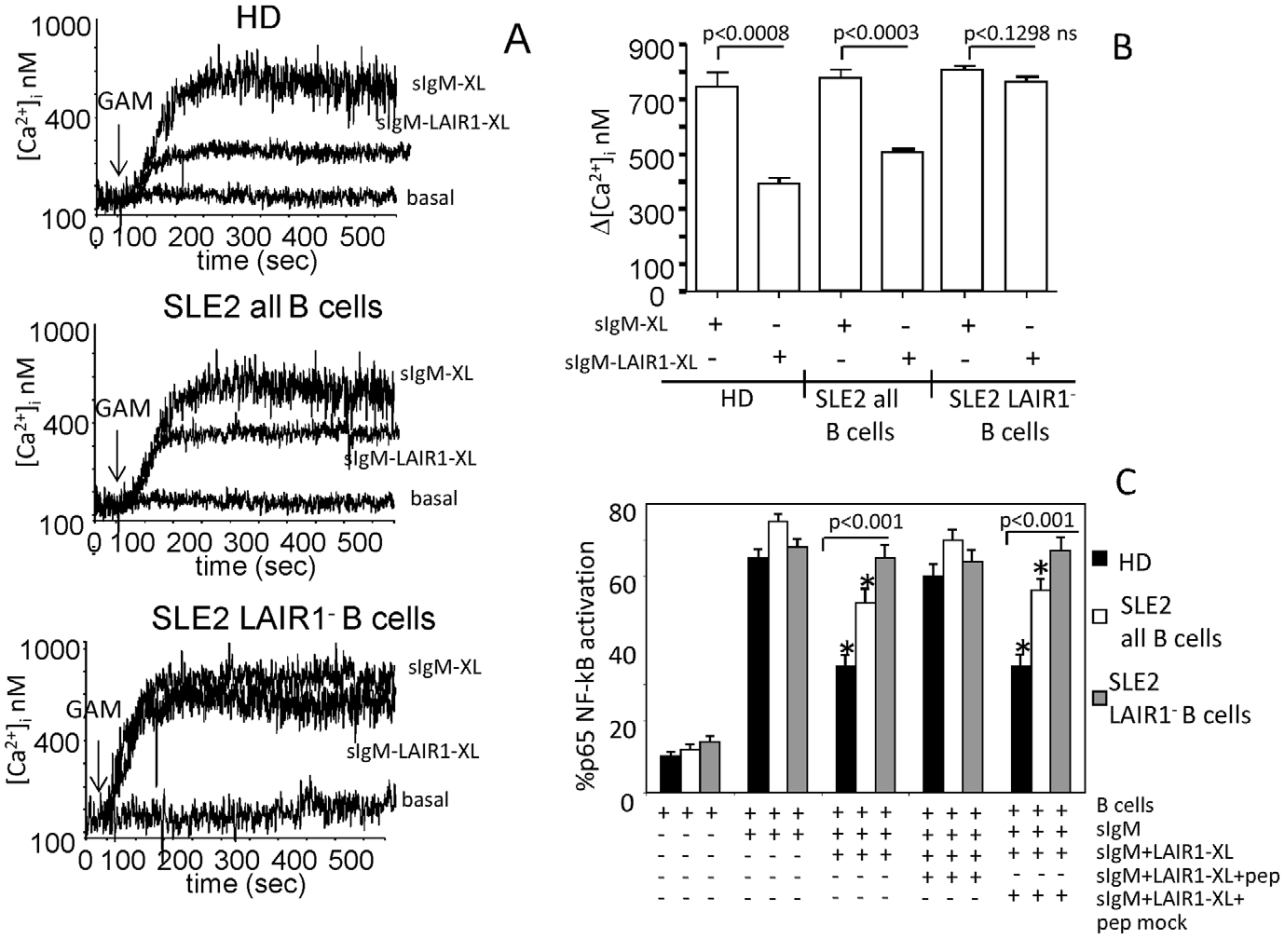
Regulation of BCR-induced calcium mobilization and activation of p65 NF-kB subunit by co-engagement of LAIR1 is defective in SLE patients. A and B. Purified B cells obtained from HD (n = 4) or SLE2 (n = 4), as indicated, were loaded with fura-2AM (1 µ M), and [Ca^2+^]_i_ increase was analyzed by monitoring fluorescence with an LS-50B spectrofluorimeter. In some experiments LAIR1^−^ B (>95%) cells from SLE2 patients were obtained from the whole B cell population after negative selection using magnetic beads coated with GAM. [Ca^2+^]_i_, was measured upon cross-linking of sIgM (IgM-XL), obtained with the specific purified mAb followed by (GAM Ig), or upon co-engagement of sIgM and LAIR1 (sIgM-LAIR1-XL), as indicated. B. Δ [Ca^2+^]_i_, mean±SD from 4HD or 4 SLE2 (unfractionated B cells or LAIR1^−^ B cells. C. B cells were separated from either HD (n = 6) or SLE patients (n = 5) or CD20^+^LAIR1^−^ cells from SLE (n = 3) alone or stimulated through BCR (with sIgM antibody, 5 µ g/ml) for 12 h and then nuclear lysates were analyzed for the activation of NF-kB subunit p65. Activation of p65 was determined by evaluating the % of p65 present in nucleus extracts and total p65 protein for each sample. In some experiments, cells were pre-incubated for 30 min in the presence or absence of the SHP-1- binding peptide (pep) or the scrambled peptide (pep-mock) and cross-linking of LAIR1 (LAIR1mAb-XL). * p<0.001 vs activation of p65 NF-kB upon sIgM engagement (BCR-mediated stimulation). The statistical significance between the p65 activation in HD and SLE patients upon different culture condition is shown in the panel.

We next analyzed whether LAIR1 engagement interferes with early BCR-mediated signaling events, such as calcium mobilization and NF-kB nuclear translocation. First, we found that LAIR1 engagement could reduce BCR-induced calcium mobilization obtained upon sIgM cross-linking in B lymphocytes from HD (fig. 5A, upper panel and fig. 5B); this inhibition was defective in SLE patients (fig. 5A central panel and fig. 5B) and absent in LAIR1 negative B cells fractionated from SLE2 patients (fig. 5A lower panel and fig. 5B). Furthermore, the NF-kB subunit translocation induced through BCR engagement is less inhibited in SLE2 patients which express low levels of LAIR1. The p65 NF-kB subunit activation (i.e. its nuclear translocation) was evaluated in nuclear extracts obtained from HD B lymphocytes (n = 6) or from SLE patients (n = 5) whole B cell population (i.e. CD20^+^LAIR1^+^ and CD20^+^LAIR1^−^) before or after cross-linking of BCR alone or with LAIR1 engagement (fig. 5C). A strong p65 nuclear translocation was induced upon sIgM engagement in HD similar to that observed in SLE patients (from basal level of 10–15% to 65–80%) In B cells of HD, LAIR1 oligomerization was able to inhibit the p65 subunit NF-kB activation induced via sIgM engagement from 65% to 35%. On the other hand, LAIR1 engagement inhibits at a lower extent BCR-induced NF-kB activation on B cells of SLE patients compared to HD (degree of p65 activation in HD 35% vs 55% in SLE, p<0.01). Finally, we isolated CD20^+^LAIR1^−^ B cells from SLE patients and we found that BCR mediated p65 subunit NF-kB activation was not inhibited by LAIR1 engagement (fig. 5C). Importantly, pre-incubation of B cells with the SHP-1-binding peptide of ITIM domain of LAIR1 abrogated the inhibiting effect mediated through LAIR1 engagement in HD and SLE patients, whereas the scrambled peptide, used as a control, did not affect the LAIR1-mediated inhibiting signal (fig. 5C). These peptides did not affect the response of CD20^+^LAIR1^−^ B cells of SLE patients.

## Discussion

Herein, we have shown that in patients suffering from SLE the inhibiting surface receptor LAIR1 is expressed on a smaller fraction of CD20^+^ B cells than in healthy donors. This decrease in CD20^+^LAIR1^+^ B cells is not related to corticosteroid treatment and it appears as a characteristic feature of SLE but not of RA or SSc. Two groups of SLE patients were identified on the basis of the amount of CD20^+^LAIR1^−^ B cells and the level of expression of LAIR1 on CD20^+^LAIR1^+^ B cells. Indeed, the SLE1 subgroup displayed LAIR1 expression similar to HD, whereas the SLE2 group, that includes the majority of SLE patients, showed a low level of LAIR1 expression and a high fraction of CD20^+^LAIR1^−^ B cells. The inhibiting signal delivered through the engagement of LAIR1 by collagen or collagen-producing cells is not fully functional in B cells in SLE2 patients suggesting that in these patients the B cells Ig-producing capacity cannot be adequately regulated by the interaction of LAIR1 with collagen or with collagen-producing LMSC. This suggests that in SLE the defective LAIR1 expression on B cells may leave these cells to be fully stimulated by still undefined polyclonal stimuli and/or auto-antigens and, then, to proliferate and produce potentially pathogenic Ig, without the negative regulation initiated upon collagen-LAIR1 interaction. Interestingly, we found that LAIR1 in a subgroup of SLE patients was expressed on B cells at similar level than in healthy donors. Indeed, the engagement of LAIR1 in these patients led to a reduced synthesis of different Ig isotypes similarly to what observed in healthy B cells. On the other hand, the lower expression or the lack of LAIR1 in the other subgroup of SLE patients resulted in the strong reduction of LAIR1-mediated Ig synthesis inhibition. These findings would suggest that LAIR1 can mediate an inhibiting signal only when expressed as in healthy B cells and there is a threshold for giving the inhibiting signal as well. A possible limitation of our study is that we did not analyze the expression of LAIR1 on B cells during the acute phase of the SLE disease in a high number of patients and after therapy. Indeed, all our patients were recruited in a remission phase of the disease in the absence of evident symptoms. SLE is a pleiotropic disease that may hit several organs and the clinical manifestation are quite variegated [25]–[29]. Each patient may respond to a complex therapy with different immunosuppressant in a characteristic manner [26]–[30]. We choose patients mostly with no evident disease activity because we try to find a difference in LAIR1 expression and function between healthy individuals and SLE affected patients, avoiding the contribution of other elements due to the flare of the illness and therapy. Nevertheless, we analyzed a limited number of patients with active disease and found a reduced expression of LAIR1 on B cells, compared with HD, without an evident increase in the percentage of CD20^+^LAIR1^−^ cells.

We have recently reported that LAIR1 is downregulated in B cells of chronic lymphocytic leukaemia (CLL) [15]. Interestingly, in CLL the engagement of LAIR1 with specific mAbs blocks the constitutive and BCR-mediated Akt phosphorylation and NF-kB nuclear translocation, leading to inhibition of B cell proliferation. Importantly, this inhibiting effect was not evident in LAIR1^−^ CLL cells [15]. In this report, we show that the oligomerization of LAIR1 on whole peripheral blood B cell population, but not in LAIR1^−^ B cells, can affect BCR-mediated calcium mobilization, in keeping with a previous report [8], and NF-kB p65 nuclear translocation. It is known that BCR induced calcium mobilitation, and the following activation cascade, is dysregulated in B cells from SLE patients [30]; one possible regulating mechanism missing in SLE B lymphocytes might be related to the lower or absent expression of LAIR1 and of its ITIM domain. Another ITIM-dependent dysregulation of BCR-induced B cell activation in SLE patients might be related to a deficient FcγIIbR mediated suppression; indeed, dysfunction of this low affinity Ab receptor, which is equipped with a cytoplasmic ITIM like LAIR1, is associated with autoimmunity and it has been described in SLE patients [31]. Taken together these findings, we would suggest that the inhibition of activation of NF-kB is a key point of the LAIR1-mediated regulation of B cell response. In addition, Akt and NF-kB are targets for the action of LAIR1 also in primary myeloid leukemias [32], [33] indicating that anywhere LAIR1 is expressed, its engagement evokes a similar cellular response. We have found that LAIR1 is expressed on the majority of naïve and memory B cells. The discrepancy of this result with the reported down-regulation of LAIR1 on memory and activated B cells [8] may be dependent on the different anti-LAIR1 mAb used in these studies. In addition, we cannot exclude that our anti-LAIR1 mAb can recognize the different isoforms of LAIR1 that can be expressed on lymphocytes [2]. It is conceivable that in SLE patients would be present the auto-antigen(s) which is responsible for the disease, thus the observed downregulation of LAIR1 found in SLE patients and the strong increment in CD20^+^LAIR1^−^ B cell fraction in a subgroup of patients would be linked to an in vivo downregulation of LAIR1 upon B cell activation. In this context, we detected a statistically significant downregulation of LAIR1 upon stimulation of healthy B cells with BCR, but this downregulation was not so evident stimulating B cells with polyclonal mitogens. In addition, we did not detect a shift from LAIR1^+^ to LAIR1^−^ B cells upon in vitro stimulation. This finding would suggest that the lack of LAIR1 on a fraction of SLE B cells is not strictly linked to in vivo activation. Also steroid therapy may have a role in downregulating LAIR1 expression. Indeed, the large majority of SLE patients were under steroid therapy (table 1), however, we found a strong heterogeneity of LAIR1 expression among SLE patients treated with steroids and there was no correlation between steroid therapy and LAIR1 expression. In addition, we found that the percentage of CD20^+^LAIR1^−^ B cells were different from those found in healthy donors also in steroid untreated patients. This would suggest that steroid do not play a key role in the observed downregulation of LAIR1 antigen on SLE B cells.

Recently, it has been reported that LAIR1 expression is lower on plasmacitoid dendritic cells (PDC) in SLE pediatric patients compared to that found in healthy donors [34]. This would indicate that the downregulation of LAIR1 in SLE patients can affect cell populations which play a key role in regulating immune response. In this context, it would be of interest to analyze whether PDC can further increment B cell response and whether the lower expression of LAIR1 on both PDC and B cells can result in a lower inhibiting signal mediated through LAIR1 and collagen-producing MSC in lymph node and/or bone marrow. In conclusion, this work points out the relevance of the lack of LAIR1-mediated inhibiting signal upon interaction with collagen produced in the microenvironment by stromal cells as a trigger for the dysregulation of antibody production in SLE patients.
