# Correction: Defective Expression and Function of the Leukocyte Associated Ig-like Receptor 1 in B Lymphocytes from Systemic Lupus Erythematosus Patients

**DOI:** 10.1371/annotation/1ada33bd-76c7-4b66-906f-950b383111e8

**Published:** 2012-06-07

**Authors:** Barbara M. Colombo, Paolo Canevali, Ottavia Magnani, Edoardo Rossi, Francesco Puppo, Maria Raffaella Zocchi, Alessandro Poggi

Figure 1 is incorrect. The correct Figure 1 can be viewed here: 

**Figure:**
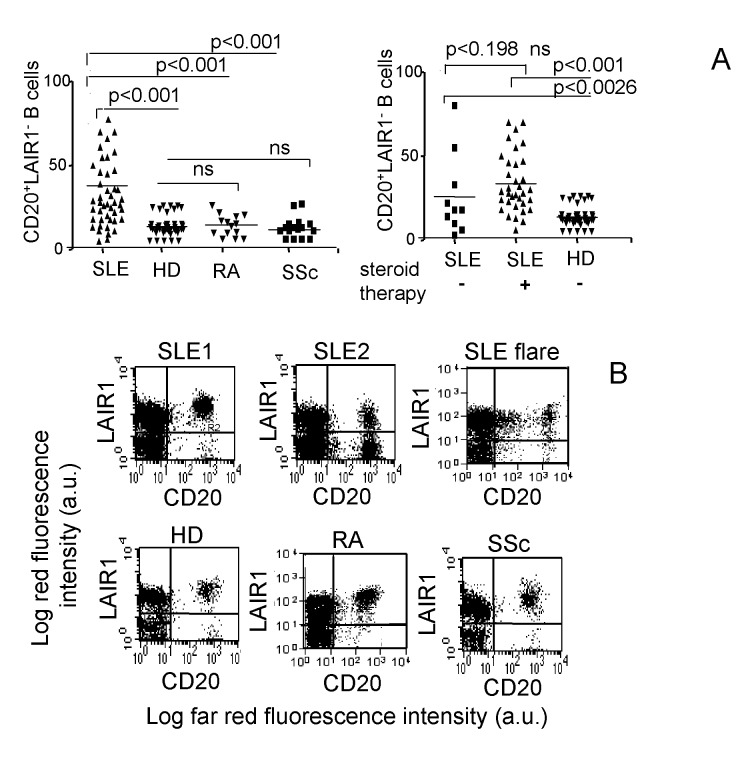


[^] 

